# Pulmonary function and fuel use: A population survey

**DOI:** 10.1186/1465-9921-6-127

**Published:** 2005-10-31

**Authors:** Asim Saha, N Mohan Rao, PK Kulkarni, PK Majumdar, HN Saiyed

**Affiliations:** 1 Occupational medicine Division, National Institute of Occupational Health, Ahmedabad – 380 016, India; 2 Respiratory Physiology Division, National Institute of Occupational Health, Ahmedabad – 380 016, India; 3 Biostatistics Division, National Institute of Occupational Health, Ahmedabad – 380 016, India; 4 Occupational medicine Division, National Institute of Occupational Health, Ahmedabad – 380 016, India; 5 Director, National Institute of Occupational Health, Ahmedabad – 380 016, India

**Keywords:** Biomass fuels, Liquefied Petroleum Gas, Pulmonary function

## Abstract

**Background:**

In the backdrop of conflicting reports (some studies reported adverse outcomes of biomass fuel use whereas few studies reported absence of any association between adverse health effect and fuel use, may be due to presence of large number of confounding variables) on the respiratory health effects of biomass fuel use, this cross sectional survey was undertaken to understand the role of fuel use on pulmonary function.

**Method:**

This study was conducted in a village of western India involving 369 randomly selected adult subjects (165 male and 204 female). All the subjects were interviewed and were subjected to pulmonary function test. Analysis of covariance was performed to compare the levels of different pulmonary function test parameters in relation to different fuel use taking care of the role of possible confounding factors.

**Results:**

This study showed that biomass fuel use (especially wood) is an important factor for deterioration of pulmonary function (particularly in female). FEV_1 _(p < .05), FEV_1 _% (p < .01), PEFR (p < .05) and FEF_25–75 _(p < .01) values were significantly lower in biomass fuel using females than nonusers. Comparison of only biomass fuel use vs. only LPG (Liquefied Petroleum Gas) use and only wood vs. only LPG use has showed that LPG is a safer fuel so far as deterioration of pulmonary function is concerned. This study observes some deterioration of pulmonary function in the male subjects also, who came from biomass fuel using families.

**Conclusion:**

This study concluded that traditional biomass fuels like wood have adverse effects on pulmonary function.

## Background

Exposure to indoor air pollution from the combustion of traditional biomass fuels (wood, charcoal, animal dung, and crop wastes) and coal is a significant public health hazard predominantly affecting poor rural and urban communities in developing countries. Large numbers of people are exposed on a daily basis to harmful emissions and other health risks from biomass and coal burning. It is estimated that globally 2.5 to 3 billion people rely on these fuels for everyday household energy needs [1]. The majority of those exposed are women, who are normally responsible for food preparation and cooking, and infants/young children who are usually with their mothers near the cooking area. Although the fraction of global energy from biofuels has fallen from 50 percent in 1900 to around 13 percent currently, this trend has leveled off and there is evidence that biofuel use is increasing among the poor in some parts of the world [1,2]. There is consistent evidence that exposure to biomass smoke increases the risk of a range of common and serious diseases of both children and adults. Chief amongst these are acute lower respiratory infections (ALRI) in childhood, particularly pneumonia [3,4]. Association of exposure with chronic bronchitis and chronic obstructive pulmonary disease is also quite well established, particularly among women [5,6]. In addition there is evidence (mainly from China), that exposure to coal smoke in the home markedly increases the risk of lung cancer, particularly in women [7,8].

So far as deterioration of lung function is concerned there is growing evidence is that biomass fuel use is harmful. Studies on biomass fuel user women [9] (urban [10] and rural [11], both) have shown that they had significantly lower pulmonary function values in comparison to non-biomass fuel users. Adverse effects of biomass fuel use have been observed in female asthmatics [12] as well as in children [13] also. At the same time there are studies where no significant difference of pulmonary function values have been observed when compared between biomass and modern fuel users [14]. Confounding effect of different other factors have been stated to be responsible for this kind of conflicting findings and the need of more such studies including intervention studies have been stressed in order to gather stronger scientific evidence [14,15]. In this backdrop this study was initiated to understand the effect of fuel use on pulmonary function.

## Material and methods

This cross sectional survey was conducted in a small village of western India situated 20 kilometers away from the nearest city. This small village had total adult population of 1509. Prevalence of pulmonary function abnormality in India being 10% in adult population, we calculated the sample size for prevalence study keeping acceptable range as 6.5–13.5%. Thus, the minimum sample size for 1% level of significance was calculated as 368. We set our target as 400 persons. Selection of subjects was done from the electoral list (voters' list) of that village by using random numbers generated from Microsoft Excel software. This list being the most frequently updated and most complete list of its kind available in India was thought to be most suitable for this purpose. Among the 400 people, who were approached for study, 369 subjects participated in this study. Necessary ethical clearance was obtained from the institutional ethics committee of National Institute of Occupational Health; India prior to the initiation of this study and informed consent was taken from the concerned study subjects during the study. These subjects were interviewed with a questionnaire to obtain information about their personal characteristics including fuel use. All of them were subjected to pulmonary function test using Spirovit-sp-10 (Schiller Health Care Ltd, Switzerland) to measure the parameters like forced vital capacity (FVC), forced expiratory volume in 1 second (FEV_1_), FEV_1_% and forced expiratory flow_25–75_(FEF_25–75_). Peak Expiratory Flow Rate (PEFR) was measured using Wright's Peak Flow Meter (Clement and Clarke, UK). FVC and FEV_1 _were expressed in litres, PEFR in litres/min, FEF_25–75 _in litres/sec and FEV_1_% was presented as the ratio of FEV_1 _and FVC expressed in percentage. Initially, a descriptive analysis was done to observe the personal characteristics of the study subjects as well as to understand the distribution of different fuel use. Afterwards different pulmonary function values were compared in males and females separately with reference to their fuel use taking care of the possible confounders. Analysis was done using SPSS Release 6.1.4 software. Analysis of covariance was performed to compare the levels of different pulmonary function parameters in relation to different fuel users taking care of the role of possible confounding factors. In our study we found that our subjects were users of wood, cattle dung, coal, kerosene and liquefied petroleum gas either alone or in combination. While categorizing we initially named wood and cattle dung users as biomass fuel users. Coal, kerosene and LPG users were treated as separate groups. Afterwards, to evaluate the effects of individual fuels within the biomass fuel group, we have treated wood and cattle dung as separate groups. Four stage analysis was actually done in relation to pulmonary function values while doing ANOCOVA analysis: biomass vs. no biomass analysis, individual fuel group wise analysis, only biomass vs. only LPG (most modern fuel among the lot) analysis and only wood (most important fuel factor according to our study in relation to deterioration of pulmonary function) vs. only LPG analysis. Though individual fuel wise analysis gave us the contribution of all the fuel factors on pulmonary function, we did different sub analyses to show the one to one comparison of different fuel groups (biomass vs. no biomass) or individual fuels (wood vs. LPG). Variables like different fuels (fuel wise yes, no), smoking (ever smoker, never smoker), house type (mud made not so well ventilated, cement and brick made well ventilated) and occupation (dusty/non-dusty) were taken as categorical variables. Other variables like age (yrs), height (centimeters), weight (kilograms) and family size were taken as continuous variables. While analyzing, we accommodated all fuel types along with possible confounding variables simultaneously in the ANOCOVA model in order to examine the effect of fuel variables adjusting for the effects of other variables. Analysis was done separately for male and female subjects.

## Results

Mean age of the study subjects was 35.5 (± 14.6) years for females (n = 204) and 38.7 (± 16.9) years for males (n = 165). In case of female subjects almost 14% were <20 years old, 103 (50.5%) subjects were in 20–39 years age group, 51(25%) subjects in 40–59 years age group and 10.8% subjects were more than 59 years old. In case of male subjects almost 13% were <20 years old, 67 (40.6%) subjects were in 20–39 years age group, 47 (28.5%) subjects in 40–59 years age group and 18.2% subjects were more than 59 years old. Mean height was 164.8 cms (± 8.9) for male subjects and 153.2 cms (± 6.4) for female subjects. Similarly mean weight was 51.9 kg (± 11.8) and 46.4 kg (± 9.9) for male and female subjects respectively.

So far as fuel use is concerned, 145 (87.9%) male and 192 (94.1%) female subjects were using biomass (wood, cattle dung) fuel either alone or in combination with other fuels. Liquefied Petroleum Gas, kerosene (a petroleum product widely used in India as a cooking fuel. It is not as purified as LPG), coal, wood and cattle dung was being used by 60 (36.4%), 58 (35.2%), 2 (1.2%), 144 (87.3%) and 16 (9.7%) male subjects respectively either alone or in combination. For female subjects the numbers were 52 (25.5%), 78 (38.2%), 7 (3.4%), 192 (94.1%) and 36 (17.6%) respectively. Only LPG was used by 14 (8.5%) male and 8 (3.9%) female subjects. Similarly wood alone was used by 63 (38.4) male and 72 (35.3%) female subjects and biomass fuel alone was used by 68 (41.4%) male and 90 (44.1%) female subjects (Table 1). All the subjects were using these fuels for more than 10 years. Cooking time was 2–3 hours/day for all the subjects.

**Table 1:** Distribution of fuel use among the study subjects

**Fuel Use**	**Male (N = 165)**	**Female (N = 204)**
Either alone or in combination		
Biomass (Wood/Animal dung)	145 (87.9)	192 (94.1)
Liquefied Petroleum Gas	60 (36.4)	52 (25.5)
Kerosene	58 (35.2)	78 (38.2)
Coal	2 (1.2)	7 (3.4)
Wood	144 (87.3)	192 (94.1)
Animal dung	16 (9.7)	36 (17.6)
**Only Biomass**	68 (41.4)	90 (44.1)
**Only liquefied petroleum gas**	14 (8.5)	8 (3.9)
**Only wood**	63 (38.4)	72 (35.3)

Table 2 shows the results of ANOCOVA analysis, where comparison of different pulmonary function parameters of female study subjects have been done in relation to different fuel use. Adjustment has been done for age, height, weight, house type, family size and occupation while doing this analysis. In individual fuel wise analysis, it was observed that pulmonary function values were comparatively lower in most of the cases in the users of wood, animal dung, coal and kerosene whereas the values were comparatively higher in case of LPG users in comparison to their respective non-users. When biomass users (either alone or in combination) were compared with non-users similar observations were found. In comparison between only wood and only LPG users, it was found that only wood users were having lower values then only LPG users. Similar findings were observed in comparison between only biomass fuel users and only LPG users. The values of FEV_1_, PEFR and FEF_25–75 _were significantly less (p < .01 in FEV_1 _and p < .05 in others) in wood users than the respective non-users. When biomass users (either alone or in combination) were compared with non-users, values of FEV_1 _(p < .05), FEV_1_% (p < .01), PEFR (p < .05) and FEF_25–75 _(p < .01) were significantly less in biomass fuel users. Similarly, only biomass fuel users were having significantly lower values of FEV_1_% (p < .01), PEFR (p < .05) and FEF_25–75 _(p < .01) in comparison to only LPG users and only wood users were having significantly lower values of FEV_1_% (p < .001), and FEF_25–75 _(p < .001) in comparison to only LPG users. The difference of PEFR values in the later case was also nearly significant (p = 0.055) statistically.

**Table 2:** Distribution of pulmonary function values of the female study subjects according to fuel use

**Fuel**	**FVC**	**FEV**_1_	**FEV**_1_**%**	**PEFR**	**FEF**_25–75_
Wood					
- Non-user	2.45 ± 0.44	2.26 ± 0.46	91.99 ± 3.98	403.50 ± 59.05	3.06 ± 0.84
- User	2.28 ± 0.62	1.97 ± 0.59**	86.44 ± 8.88	347.91 ± 80.96*	2.40 ± 0.95*
Animal dung					
- Non-user	2.31 ± 0.62	2.00 ± 0.55	86.70 ± 8.34	353.24 ± 81.02	2.45 ± 0.94
- User	2.18 ± 0.55	1.89 ± 0.49	87.09 ± 10.67	341.58 ± 80.14	2.42 ± 1.05
Coal					
- Non-user	2.29 ± 0.62	1.99 ± 0.55	86.77 ± 8.84	351.61 ± 81.54	2.45 ± 0.96
- User	2.22 ± 0.35	1.93 ± 0.39	86.61 ± 6.72	339.00 ± 59.02	2.30 ± 0.77
Kerosene					
- Non-user	2.30 ± 0.65	2.02 ± 0.59	87.58 ± 8.44	353.52 ± 87.19	2.53 ± 1.01
- User	2.27 ± 0.54	1.93 ± 0.46	85.46 ± 9.17	347.40 ± 69.60	2.30 ± 0.85*
LPG					
- Non-user	2.26 ± 0.64	1.96 ± 0.56	86.61 ± 8.75	347.53 ± 83.70	2.38 ± 0.96
- User	2.36 ± 0.52	2.06 ± 0.49	87.23 ± 8.87	361.86 ± 71.32	2.62 ± 0.99
Biomass					
- Non-user	2.45 ± 0.44	2.26 ± 0.46	91.99 ± 3.98	403.50 ± 59.05	3.06 ± 0.84
- User	2.28 ± 0.62	1.97 ± 0.54*	86.44 ± 8.89**	347.91 ± 80.96*	2.40 ± 0.95**
Only biomass user	2.25 ± 0.68	1.96 ± 0.61	86.90 ± 8.92**	345.50 ± 93.36*	2.41 ± 1.00**
Only LPG user	2.45 ± 0.52	2.29 ± 0.55	93.15 ± 4.09	407.50 ± 71.94	3.22 ± 0.97
Only wood user	2.29 ± 0.70	1.98 ± 0.63	86.13 ± 8.66***	347.55 ± 97.04	2.37 ± 0.98***
Only LPG user	2.45 ± 0.52	2.29 ± 0.55	93.15 ± 4.09	407.50 ± 71.94	3.22 ± 0.97

* p < 0.05

** p < 0.01

*** p < 0.001

Adjusted for age, height, weight, house type, family size and occupation.

Table 3 shows the comparison of pulmonary function values in case of male subjects. In this case the difference of pulmonary function values were not so prominent as in case of female study subjects. Though the pulmonary function values were not significantly different, while comparing different fuel users with their respective non-users in individual fuel wise analysis, some of the values showed significant difference when only biomass fuel users were compared with only LPG users and when only wood users were compared with only LPG users. FEV_1 _(p < .05), PEFR (p < .05) and FEF_25–75 _(p < .05) values were significantly lower in only biomass fuel users as well as in only wood users when compared with only LPG users.

**Table 3:** Distribution of pulmonary function values of the male study subjects according to fuel use

**Fuel**	**FVC**	**FEV**_1_	**FEV**_1_**%**	**PEFR**	**FEF**_25–75_
Wood					
- Non-user	3.33 ± 0.81	2.74 ± 0.76	81.83 ± 9.02	453.40 ± 117.79	2.88 ± 1.04
- User	2.98 ± 0.87	2.48 ± 0.80	82.68 ± 10.20	427.11 ± 122.42	2.71 ± 1.15
Animal dung					
- Non-user	3.03 ± 0.88	2.52 ± 0.81	82.55 ± 10.25	431.89 ± 125.79	2.75 ± 1.16
- User	2.94 ± 0.81	2.44 ± 0.70	82.79 ± 8.09	415.75 ± 104.06	2.58 ± 0.94
Coal					
- Non-user	3.02 ± 0.87	2.51 ± 0.80	82.42 ± 10.00	429.38 ± 122.28	2.72 ± 1.14
- User	2.64 ± 0.53	2.50 ± 0.42	95.04 ± 3.28	506.00 ± 1.41	3.62 ± 0.35
Kerosene					
- Non-user	2.96 ± 0.93	2.47 ± 0.85	82.83 ± 9.68	422.65 ± 130.64	2.72 ± 1.20
- User	3.13 ± 0.75	2.58 ± 0.69	82.12 ± 10.74	444.33 ± 103.40	2.76 ± 1.02
LPG					
- Non-user	2.93 ± 0.92	2.45 ± 0.85	82.72 ± 10.41	418.27 ± 128.01	2.71 ± 1.22
- User	3.17 ± 0.74	2.62 ± 0.68	82.32 ± 9.45	451.20 ± 108.14	2.78 ± 0.99
Biomass					
- Non-user	3.33 ± 0.81	2.74 ± 0.76	81.83 ± 9.02	453.40 ± 117.79	2.88 ± 1.04
- User	2.98 ± 0.87	2.48 ± 0.79	82.64 ± 10.17	427.50 ± 122.09	2.71 ± 1.15
Only biomass user	2.87 ± 0.98	2.40 ± 0.91*	82.76 ± 10.46	411.28 ± 142.02*	2.70 ± 1.29*
Only LPG user	3.36 ± 0.93	2.74 ± 0.88	80.77 ± 10.25	440.86 ± 128.32	2.88 ± 1.19
Only wood user	2.84 ± 1.01	2.38 ± 0.94*	82.51 ± 10.80	407.14 ± 145.33*	2.67 ± 1.32*
Only LPG user	3.36 ± 0.93	2.74 ± 0.88	80.77 ± 10.25	440.86 ± 128.32	2.88 ± 1.19

* p < 0.05

Adjusted for age, height, weight, smoking, house type, family size and occupation.

## Discussion

In a study conducted in Turkey a highly significant (p < 0.00001) reduction of FEV_1_, FVC, FEV_1_/FVC and FEF_25–75 _was observed in case of biomass fuel users [9]. A study conducted in an urban Indian slum showed significantly lower FVC, FEV_1_, FEV_1_% and PEFR values in bio-fuel using women in comparison to modern fuel users (kerosene and LPG) [10] whereas a similar study undertaken involving rural Indian women could show the prominent adverse effect of biomass fuel use on FVC only [11]. This deterioration of pulmonary function in biomass fuel users has been attributed to the fact that the amount and concentration of particulate matter and other toxic gases emitted during biomass combustion while cooking are more than those emitted during combustion of LPG [16].

This study has come up with the finding that biomass fuel use (especially wood use) is an important factor for deterioration of pulmonary function. Wood, animal dung, coal and kerosene users (female) were having comparatively lower pulmonary function values than their respective non-users whereas LPG users were having comparatively higher values. Comparison of only biomass fuel vs. only LPG use and only wood use vs. only LPG use in females has showed that LPG is better than others so far as deterioration of pulmonary function is concerned. The pulmonary function parameters affected by biomass fuel use have been FEV_1_, FEV_1_%, PEFR and FEF_25–75_. No effect has been observed on FVC. This study has also observed some effect on the male members of biomass using families (especially wood) while comparing only biomass fuel vs. only LPG use and only wood use vs. only LPG use.

Most of the families being farmers, cooking is usually done in early morning or evening hours to enable both male and female members to go to field for agricultural activities. Otherwise also, cooking in such hours was found to be the custom of this village and thereby both the male and female members of the families were exposed to the effects of cooking fuels because of their presence during cooking hours. However, direct involvement to the act of cooking may have been the reason for more exposure in females, which may have caused more effects in females.

This study has made an effort to derive the effects of different fuel use on pulmonary function adjusting for the effects of the confounders as much as possible. Adjustment of the effect of confounders being extremely important [14,15] in such a study, this effort has been done as meticulously as possible. The effects of age, height, weight, house type (ventilation status), family size (overcrowding) and occupation have been adjusted for. All the subjects reported that they were using the fuel for more than last 10 years and the cooking time/day was 2–3 hours. Lack of specific information on exact cooking time and exact duration of fuel used restricted us from examining the effect of these two variables as confounders. This has been a limitation of this study. Inclusion of large number of variables in the ANOCOVA analysis model may have been another limitation of this study considering its sample size (calculated sample size was for a prevalence study only). With a larger sample size in addition to the effects of individual fuels, effects of all possible fuel combinations could have been studied. Nevertheless, this study has included the combination fuel users also in the analysis (unlike previously reported studies) along with single fuel users and the effect of fuel use on pulmonary function has been estimated not only by comparing individual fuel users vs. non-users but also with the one to one comparison of different fuel users. This study being a cross sectional one also bears the restriction of understanding the temporal relationship of fuel use and pulmonary function deterioration. Selection of subjects from more villages also could have made the findings of this study more generalisable.

## Conclusion

This study eventually concludes showing the adverse effects of biomass fuels (especially wood) use on the deterioration of pulmonary function. The findings of this study point towards an important environmental health problem involving mostly the poor women and the children and indicate that the health consequences of exposure from biomass and other solid fuels in developing countries should not be ignored not only because the health burden is high but also because of the fact that such fuels will continue to be used throughout the world by a large number of households in the foreseeable future because of economic reasons.

## Competing interests

The author(s) declare that they have no competing interest.

## Authors' contributions

**AS: **Planned, executed the study. Prepared the write up.

**NMR: **Involved in planning and execution of the study.

**PKK: **Performed statistical analysis

**PKM: **Executed the study, helped in literature search.

**HNS: **Guided in planning, executing the study and in preparing the write up.
